# Development of multiplex real-time RT-PCR assay for the detection of SARS-CoV-2

**DOI:** 10.1371/journal.pone.0250942

**Published:** 2021-04-29

**Authors:** Huseyin Tombuloglu, Hussein Sabit, Ebtesam Al-Suhaimi, Reem Al Jindan, Khaled R. Alkharsah

**Affiliations:** 1 Department of Genetics Research, Institute for Research and Medical Consultations (IRMC), Imam Abdulrahman Bin Faisal University, Dammam, Saudi Arabia; 2 Department of Biology, College of Science and Institute for Research and Medical Consultations (IRMC), Imam Abdulrahman Bin Faisal University, Dammam, Saudi Arabia; 3 Department of Microbiology, College of Medicine, Imam Abdulrahman Bin Faisal University, Dammam, Saudi Arabia; Dokuz Eylul Universitesi, TURKEY

## Abstract

The outbreak of the new human coronavirus SARS-CoV-2 (also known as 2019-nCoV) continues to increase globally. The real-time reverse transcription polymerase chain reaction (rRT-PCR) is the most used technique in virus detection. However, possible false-negative and false-positive results produce misleading consequences, making it necessary to improve existing methods. Here, we developed a multiplex rRT-PCR diagnostic method, which targets two viral genes (*RdRP* and *E*) and one human gene (*RP*) simultaneously. The reaction was tested by using *pseudoviral* RNA and human target mRNA sequences as a template. Also, the protocol was validated by using 14 clinical SARS-CoV-2 positive samples. The results are in good agreement with the CDC authorized Cepheid`s Xpert^®^ Xpress SARS-CoV-2 diagnostic system (100%). Unlike single gene targeting strategies, the current method provides the amplification of two viral regions in the same PCR reaction. Therefore, an accurate SARS-CoV-2 diagnostic assay was provided, which allows testing of 91 samples in 96-well plates in per run. Thanks to this strategy, fast, reliable, and easy-to-use rRT-PCR method is obtained to diagnose SARS-CoV-2.

## Introduction

The outbreak of novel *Betacoronavirus*, SARS-CoV-2, which began in Wuhan, China in December 2019, has spread rapidly to multiple countries as a global pandemic. As of March 10, 2021, about 135 million people were confirmed with SARS-CoV-2 infection, and three million were died (https://www.worldometers.info/coronavirus/#countries). The increasing number of infections worldwide necessitates the need for a less-invasive, reliable, and fast diagnostic tool [1]. To achieve this goal, several studies have tackled this challenge and these efforts have yielded several diagnostic kits. Many approaches have been proposed to detect SARS-CoV-2 virus in nasopharyngeal fluids such as multiplex RT-PCR [2, 3], CRISPR/Cas12 [4, 5], and CRISPR-Cas3 [6], lateral flow immunoassay [7], paper-based biomolecular sensors [8], SHERLOCK testing in one pot [9], DNA aptamer [10], loop-mediated isothermal amplification (LAMP) [11], etc. Each of these methods has its own strong and weak points in terms of sensitivity and specificity. Among these methods, nucleic acid amplification-based tests are the most common for the diagnosis of SARS-CoV-2. To date, the US Food and Drug Administration (FDA) has approved 196 molecular diagnostic tests for the detection of SARS-CoV-2 nucleic acids as Emergency Use Authorization (EUA) (https://www.fda.gov/medical-devices/coronavirus-disease-2019-covid-19-emergency-use-authorizations-medical-devices/vitro-diagnostics-euas). In addition, US CDC (The Centers for Disease Control and Prevention) suggest a protocol for the detection of SARS-CoV-2, based on the amplification of two regions of the nucleocapsid gene, namely *N1* and *N2*, and a human internal control gene *RNase P* (*RP*) [12]. In addition to these strategies, efforts to develop SARS-CoV-2 detection methods with high efficiency and accuracy, with less reaction time and effort, are still ongoing.

SARS-CoV-2 is a positive-sense single-stranded RNA ((+) ssRNA) virus. Its genome consists of 29,900 nucleotides (nt) enclosing five open reading frames (ORFs) (5′–3′); ORF1ab polyprotein (P, 7,096 amino acids), spike glycoprotein (S, 1,273 amino acids), nucleocapsid protein (N, 419 amino acids), envelope protein (E, 75 amino acids), and membrane protein (M, 222 amino acids) [1, 13]. Therefore, several viral genes could be targeted for the detection of SARS-CoV-2 by RT-PCR methods. These genes include such as *RdRP* and *S genes* (Yan et al. 2020) [14], *N* and *S* genes [2], and *E* gene [15]. For the best RT-PCR performance, the combination of these potential gene targets should be optimized.

In the present study, it is aimed to develop multiplex real-time reverse transcription polymerase chain reaction (rRT-PCR) assay for the detection of SARS-COV-2. The assay simultaneously targets two viral genes (*RdRP* and *E*) and a human gene (*RP*) as an internal control by using the Applied Biosystems 7500 Fast Real-Time PCR instrument (ABI, Thermo Fisher Sci). In addition, the clinical performance of the assay was evaluated on SARS-CoV-2 samples collected from COVID-19 positive patients and compared by using the GeneXpert Dx instrument (Cepheid, Sunnyvale, CA, USA).

## Materials and methods

### Alignment of SARS-nCoV-19 genome sequences

Genomic sequences of all SARS-nCoV-19 types that have been sequenced worldwide were downloaded from the database of GISAID (Global Initiative on Sharing All Influenza Data, https://www.gisaid.org). The comparative analyses by aligning the sequences at a base level were made with bioinformatics programs such as NCBI BLAST [16], MUSCLE [17], and Clustal Omega [18]. More than 100 annotated genomes, whose genome sequence information have been determined and which include samples from Europe and Asia, were selected. In this way, virus gene targets are adjusted as sensitive, specific, and accurate as possible.

### Multiplex primer / probe design

**Fig 1:**
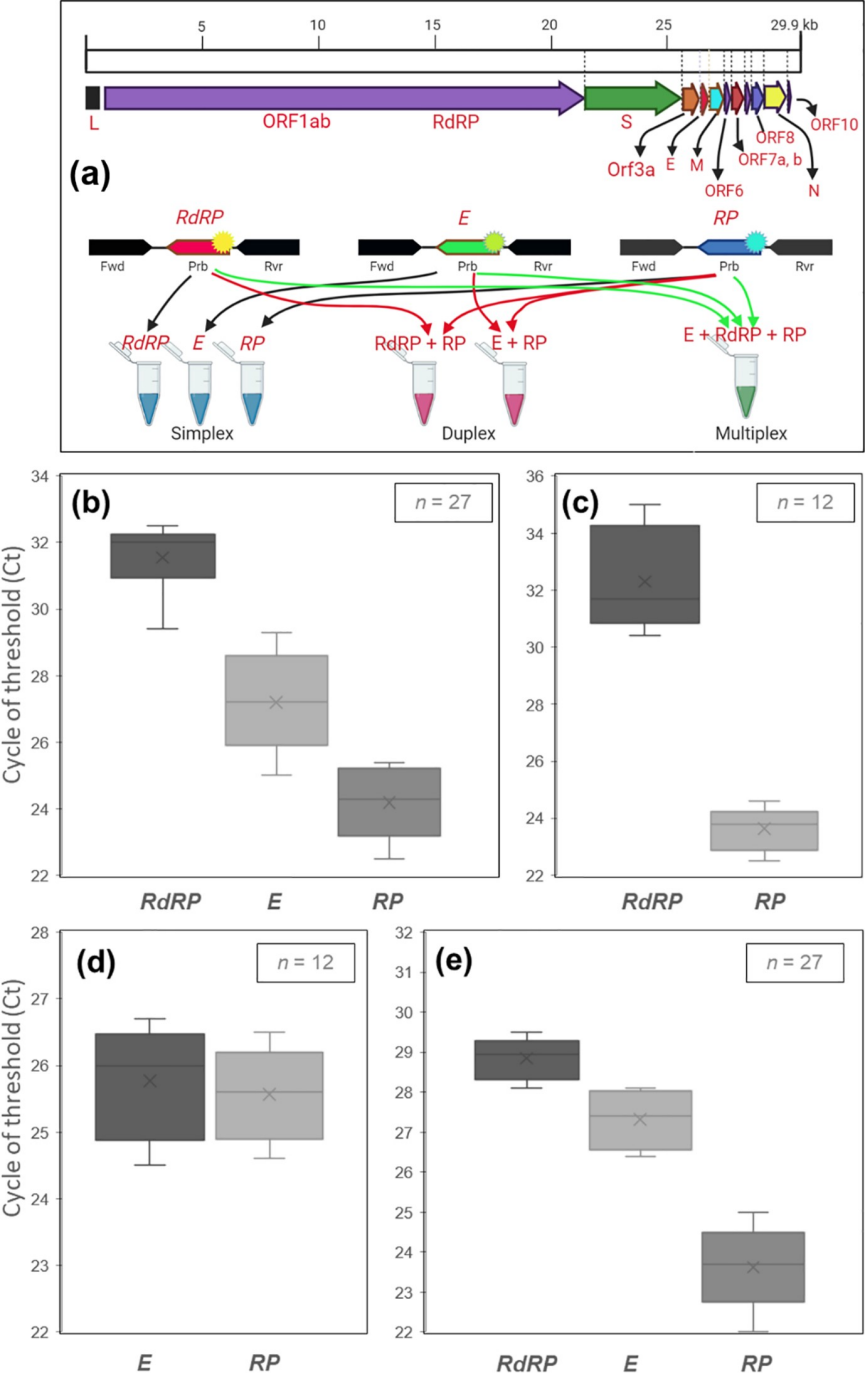
Experimental design and the cycle threshold (Ct) value of rRT-PCR repeats. **(a)** Genome organization of SARS-CoV-2 and selected gene/probe sets. **(b)** Simplex assay targeting *RdRP*, *E*, and *RP* genes in separate reaction tubes. Multiplex or duplex assay amplifies two genes simultaneously: **(c)** *RdRP* and *RP*, **(d)** *E* and *RP*. **(e)** Multiplex or triplex assay that targets two viral (*RdRP* and *E*) and one human internal control (*RP*) gene simultaneously.

Multiplex PCR compatible primer and probe arrays that are specific to viral and human gene targets were designed using programs such as PrimerPooler [19], PrimerPlex (http://www.premierbiosoft.com/primerplex/index.html), and Primer3 [20]. 5’ Fluorescein amidites (FAM) and 3’ black hole quencher-1 (BHQ-1)-labeled probe for the viral *RdRp* gene, 5’ hexachloro-fluorescein (HEX) and 3’ BHQ-1-labeled probe for the viral *E* gene, and a 5’ carboxyrhodamine (ROX) and 3’ BHQ-2-laballed probe for the human *RP* gene were designed and synthesized (Fig 1A). The concentration and sequence of each primer or probe are stated in Table 1.

**Table 1:** The sequence and concentration of primer and probe sets used in PCR reactions.

Primer/probe	Sequence (5`- 3`)	Concentration (μM)	Reference
***RdRp—*F**	GTCATGTGTGGCGGTTCACT	20	This study
***RdRp—*R**	CAACACTATTAGCATAAGCAGTTGT	20	
***RdRp-* P**	FAM-CAGGTGGAACCTCATCAGGAGATGC- BHQ1	5	[33]
***E-* F**	GGAAGAGACAGGTACGTTAATA	20	This study
***E-* R**	AGCAGTACGCACACAATCGAA	20	
***E-* P**	HEX-ACACTAGCCATCCTTACTGCGCTTCG-BHQ1	5	[32]
***RP—*F**	AGATTTGGACCTGCGAGCG	20	Universal
***RP—*R**	GATAGCAACAACTGAATAGCCAAGGT	20	
***RP—*P**	ROX-TTCTGACCTGAAGGCTCTGCGCG-BHQ2	5	

### RNA isolation

The study is approved by the Institutional Review Board (IRB) at Imam Abdulrahman bin Faisal University (IAU) with an IRB number of IRB-2020-13-406. Also, it is approved by the institutional research committee and the de-identified samples were left over after completion of diagnostic tests; hence this study requires no consenting as per institutional ethics committee regulations.

Viral RNA was extracted from nasopharyngeal swabs in virus transport medium (VTM) which were sent to the microbiology laboratory at King Fahd Hospital of the University (KFHU), Al Khobar for SARS-CoV-2 detection. RNA extraction was performed from 280 μL of the VTM using the QIAamp Viral RNA Mini kit (Qiagen, Hilden, Germany) according to the manufacturer’s instructions.

### rRT-PCR reaction

The reaction mixture (20 μL) includes the following reagents: 2 μL of 10x Buffer, 0.25 μL of dNTPs (10 mM each), 0.2 μL of uracil-DNA glycosylase (UDG) (1 U/μL), 0.4 μL of VitaTaq® HS polymerase (2 U/μL), 0.05 μL VitaScript® Enzyme mix including M-MLV (Procomcure, Austria), 0.05 μL of Triton™ X-100 (molecular biology grade, Merck), the primer and probe mixture, and RNase/DNase-free ddH_2_O up to 20 μL. The mixture for the primer and probe is varied according to the kit design. For the multiplex kit that simultaneously targeting the three genes, the final concentration of the primers/probes were adjusted as follow:

1. 10 pM for RdRP-F, 13 pM for RdRP-R, and 4 pM for RdRP-P
2. 4 pM for E-F, 4 pM for E-R, and 2 pM for E-P
3. 10 pM for RP-F, 3.75 pM for RP-R, and 4 pM for RP-P

The mixture was dispensed in 96-well plates (MicroAmp™ Fast Optical 96-well reaction Plate 0.1 mL, Applied Biosystems) and sealed with optical film (MicroAmp™ Optical Adhesive Film, Applied Biosystems). *Pseudoviral* RNAs including viral *RdRP* and *E* gene and human *RNaseP* (*RP*) mRNA sequences were used as the positive template. Meanwhile, RNase/DNase-free ddH_2_O was added to the negative control tubes to check any contamination or primer dimer.

Quantitation experiments were performed in a real-time PCR instrument (Applied Biosystems™, 7500 Fast Real-Time PCR System). Before the operation, the instrument was calibrated by using Applied Biosystems™ 7500 Fast Real-Time PCR Systems Spectral Calibration Kit. Then, the qPCR reaction conditions were adjusted as follow: 1) Reverse transcription at 45°C for 5 min, 2) Pre-denaturation at 95°C for 30 sec, 3) 40 cycles of denaturation at 95°C for 5 sec and amplification at 60°C for 30 sec. The reporter dye channel sets as FAM for viral *RdRp* gene; and VIC for *E* gene; and ROX for human *RNAseP* (*RP*) gene. For the Applied Biosystems™ real-time PCR instrument (7500 and StepOne models), set to “passive reference” dye as “none”.

### Amplification efficiency

To find out the amplification efficiency (E) of the genes, a standard curve from the dilution series of templates was prepared. Ct values versus the logarithmic amount of the template were plotted. The amplification efficiency was obtained by using the following equation:

$$E=100\;x\;\left(10^{‐1/\mathrm{slope}}\right)$$ (1)

### Validation of the assay by Xpert Xpress SARS-CoV-2

The nasopharyngeal swabs used for the validation of the current assay were also tested for SARS-CoV-2 using the Xpert Xpress SARS-CoV-2 kit (n = 14) (Cepheid, Sunnyvale, CA, USA). About 300 μL of the VTM were transferred to the Xpert Xpress SARS-CoV-2 kit cartridge. The kit includes direct RNA extraction and rRT-PCR targeting the *E* and *N2* gene fragments of the SARS-CoV-2. The assay was run on the GeneXpert Dx instrument (Cepheid, Sunnyvale, CA, USA).

### Data analysis

The results were evaluated by determining the amplification curve of the target gene and the internal control gene. For the ABI 7500 device, the cycle threshold (Ct or Cq) line was automatically adjusted to ensure that the curves are all straight position. For this purpose, ABI 7500 software (v2.3) was used. The cycle threshold number ≤ 38 with a sigmoidal curve is accepted as `positive`.

## Results

### Simplex rRT-PCR standardization

Before multiplexing, all targeted gene primer/probe set, and RT-PCR reagents were tested in simplex rRT-PCR. The quantity of SARS-CoV-2 specific *E* and *RdRP* gene primer and probe set were optimized by using 10^5^ copy/μL of synthetic viral RNA as a template. Before the reactions, the rRT-PCR instrument (Applied Biosystems™, 7500 Fast Real-Time PCR System) was calibrated to get the best fluorescent performance. The simplex reactions were repeated at least six times for two viral *E* and *RdRP* genes and one internal control (IC) gene *RP*. The average cycle threshold (Ct) value with standard deviations (SD) were 24.1 ± 1.05, 27.1 ± 1.5, and 31.5 ± 1.0 for *RP*, *E*, and *RdRP*, respectively (Fig 1B).

### Duplex rRT-PCR standardization

The primer and probe sets were designed to detect one viral (*E* or *RdRP*) and one human IC gene (*RP*) at the same rRT-PCR reaction. Two different reaction mixtures were designed including the primer and probe sets either for *RdRP* with *RP*, or *E* with *RP*. Triplicate reactions yielded the Ct value as 23.6 ± 0.77 and 32.3 ± 1.8 for *RP* and *RdRP* genes, respectively (Fig 1C). In the second reaction mixture, the Ct value was detected as 25.7 ± 0.84 and 25.5± 0.73 for *E* and *RP* genes, respectively (Fig 1D). The Ct values of both reactions are less than 38, which is the recommended limit of CDC (Center of Disease Control and Prevention).

### Multiplex (triplex) rRT-PCR standardization

Multiplex rRT-PCR protocol was set up for the amplification of three genes (*E*, *RdRP*, and *RP*). Three primer and probe set for each gene were combined in the same reaction tube. Sigmoidal amplification curves were obtained with an average Ct value ± SD as 28.8 ± 0.51, 27.3± 0.7, and 23.6± 1.04 for *RdRP*, *E*, and *RP* genes, respectively (Fig 1E). This shows that the assay is capable to detect three genes in the same reaction tube.

### Limit-of-detection (LOD) and rRT-PCR efficiency

**Fig 2:**
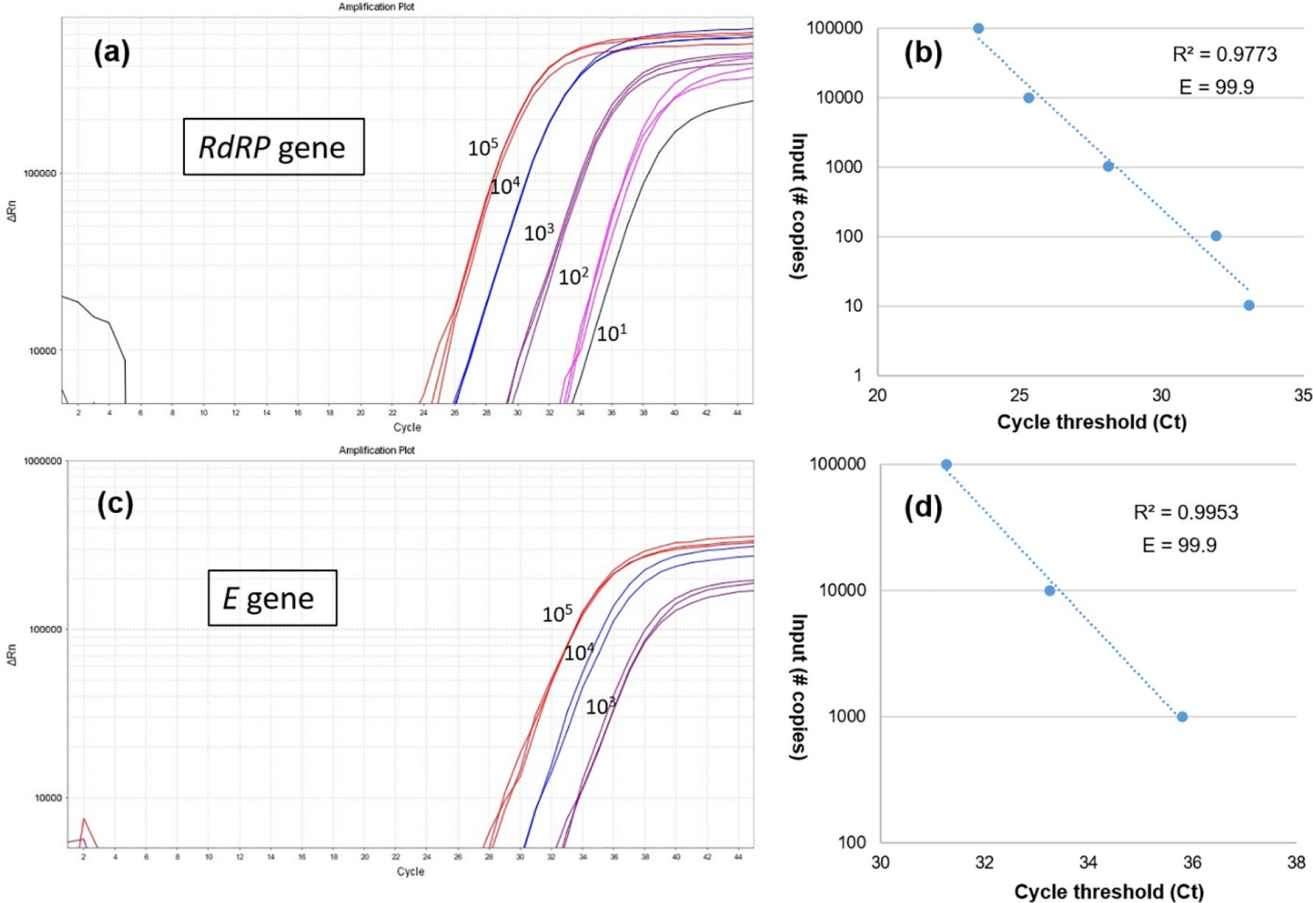
The limit-of-detection (LOD) of *RdRP* and *E* genes. A serial dilution of synthetic RNA (10^5^, 10^4^, 10^3^, 10^2^ and 10^1^ copies/ μL) was prepared. The amplification plots (**a, c**) and the amplification efficiencies (E) **(b, d)** were represented.

A serial dilution of synthetic RNA (10^5^, 10^4^, 10^3^, 10^2^ and 10^1^ copies/μL) was prepared to find the limit-of-detection (LOD) for *RdRP* and *E* genes. The amplification plots, the amplification efficiencies (E), and R^2^ score are represented in Fig 2. The LOD of *RdRP* gene was ≥10 copy/μL. Nevertheless, the LOD of *E* gene was ≥10^3^ copy/μL. The E value of *RdRP* and *E* genes were determined as 99.9. The R^2^ scores were determined as 0.977 for the *RdRP* and 0.995 for the *E* gene.

### Validation of the assay using SARS-CoV-2-positive samples detected by Cepheid`s system

**Fig 3:**
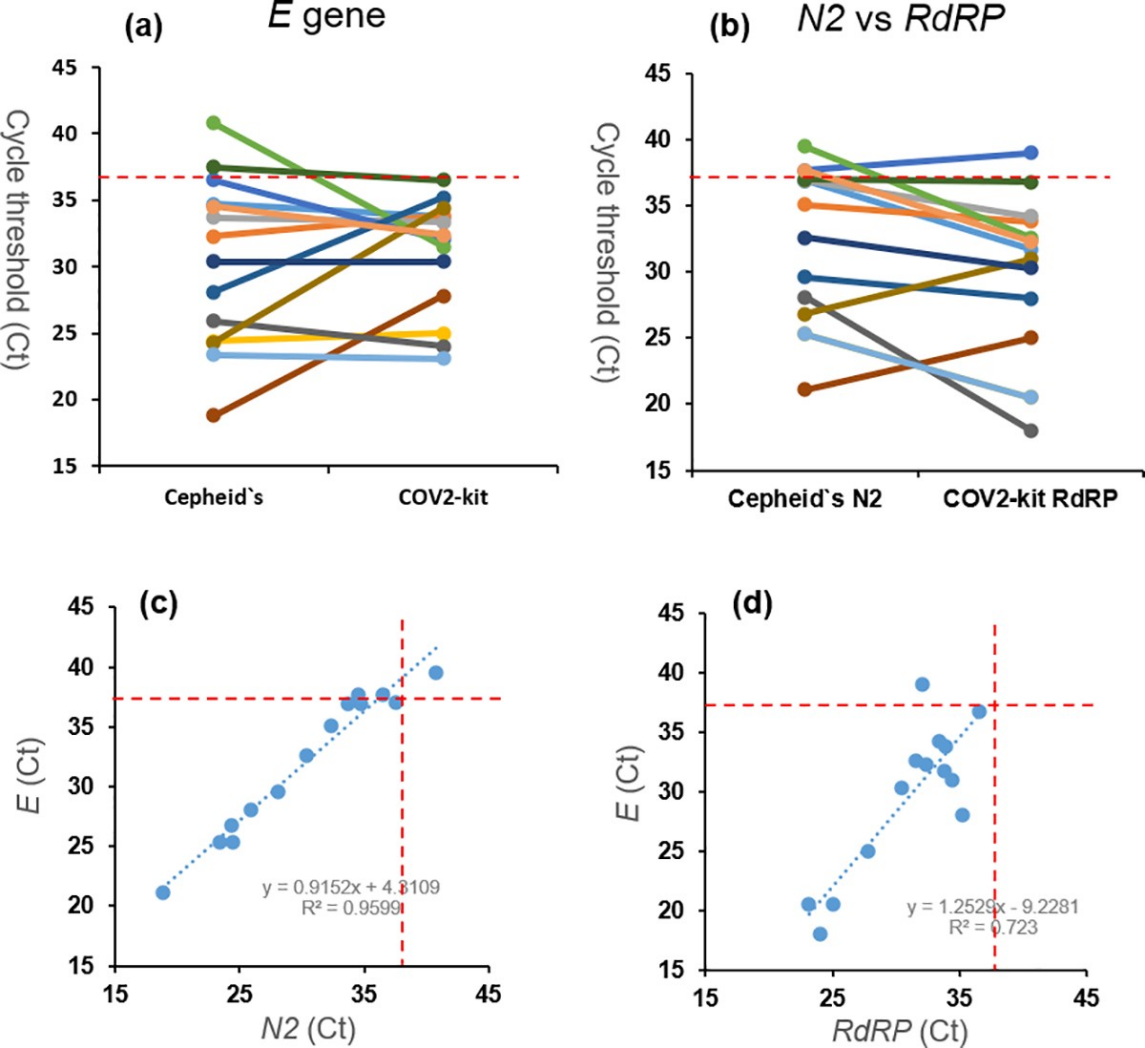
Comparison of the cycle threshold (Ct) value of COVID-19 positive samples using Cepheid`s and the current (COV2-kit) assays. **(a)** The data shows the Ct value of *E* gene, which is the common gene in both assays. **(b)** The comparison of Cepheid`s *N2* and COV2-kit`s *RdRP* genes. The plots show the comparison of Ct values of the target genes for **(c)** Cepheid`s *E* and *N2*; and **(d)** COV2-kit`s *RdRP* and *E*. The red dashed line shows threshold value of 37, which is accepted as the upper limit for SARS-CoV-2 detection by CDC.

To validate the outcome of multiplex rRT-PCR assay (named as COV2-kit) in the clinical SARS-CoV-2-positive samples, we compared our results by using Cepheid’s GeneXpert^®^ System. For this purpose, firstly the nasopharyngeal swabs were collected from COVID-19 infected patients between the 1^st^ of November and the 5^th^ of December 2020 at King Fahd Hospital of University (KFHU), Al Khobar. Then, the samples were kept in VTM medium and 300 μL of the solution was directly transferred to the Cepheid’s GeneXpert^®^ cartridge. The samples were run by using Xpert® Xpress SARS-CoV-2 detection kit. The SARS-CoV-2 positive samples were selected for viral RNA extraction (QIAamp Viral RNA Mini Kit, Qiagen, Germany). Then, the extracted RNA was used as template to test our assay. The comparative Ct performances of each assay were shown in Fig 3. The COV2-kit detected all confirmed SARS-CoV-2 positive samples. All Ct values of the *E* gene are below the threshold accepted by the CDC (≤37) (Fig 3A). For the *RdRP* gene, only one clinical sample was out of the threshold, which is consistent with Cepheid’s assay (Fig 3B). According to the comparison of Ct values between Cepheid’s *E* and *N2* (Fig 3C), and the *RdRP* and *E* genes of COV2-kit, there is agreement between the obtained data (Fig 3D).

## Discussion

The global increase in the COVID-19 pandemic makes the development of faster, reliable, and sensitive virus detection methods a priority [21]. Extensive studies to find an efficient, reliable, and sensitive detection method for SARS-CoV-2 are still ongoing. This study aims to develop a multiplex rRT-PCR test that targets two viral (*RdRP* and *E*) and one human (*RP*) gene simultaneously in a single reaction tube. The assay is named as COV2-kit. Thanks to the specific probes that were labelled with different fluorescence dyes (HEX, ROX, and FAM), the gene amplifications can be identified in the same reaction tube by using different filters of the Applied Biosystems™, 7500 Fast Real-Time PCR System. Three different approaches were performed to optimize the protocol: simplex (targets single gene), duplex (simultaneously targets two genes) and triplex (simultaneously targets three genes). For this purpose, a synthetic viral template together with mRNA of *RP* gene was used. The Ct values of the reactions are ranged in between 24 to 34. According to the CDC (Center of Disease Control and Prevention) and WHO recommendations, the samples with a Ct value of 37.01 or greater are considered as negative (CDC, https://www.cdc.gov/dengue/healthcare-providers/testing/molecular-tests/faq_rt-pcr.html; WHO, https://extranet.who.int/pqweb/sites/default/files/documents/201005_final_pqpr_eul_0517_204_00_sars_cov2_nucleic_acid_detection%20%281%29.pdf). In other words, the Ct value should not exceed 37 to accept the sample as positive. The Ct values in all tested approaches (simplex, duplex, or triplex) were under this threshold. The LOD (limit-of-detection) for *RdRP* and *E* genes were at least 10^1^ and 10^3^ copy/μL, respectively. The primers and probe for the *RdRP* gene were found to be more sensitive than these for the *E* gene. The viral load of the COVID-19 patients is the critical factor for the test efficiency [22]. It can be concluded that *RdRP* gene provides the maximum detection capability on the patients having low viral load (≥10^1^ copy/μL). In addition, the patients with a viral load of ≥10^3^ copy/μL can be detected by using the *E* gene as a target. Thus, the samples with a low viral load can be detected by using these two sensitive gene targeting approaches. In addition to these viral genes, the reaction includes a human gene target, *RP*, as an internal control (IC). In general, the IC gene is tested in a separate tube for each reaction which decreases the sample size to be tested and increase the expenses. By using the current strategy, the number of reactions per sample is reduced. For instance, the assay allows testing 91 patients in 96-well plates per run, thus provides less time and save expensive RT-PCR reagents. Fast, reliable, high-sensitivity and low-cost SARS-CoV-2 detection is achieved by designing and using effective primer / probe sets.

The COV2-kit assay was tested to verify its performance on clinically SARS-CoV-2 positive samples. In addition, the results were compared by using Cepheid’s GeneXpert^®^ System that uses Xpert® Xpress SARS-CoV-2 detection kit (Fig 3). Rather than our strategy that targets the *RdRP* and *E* genes, the Xpert® Xpress SARS-CoV-2 detects *N2* and *E* genes. The Ct value of the *E* gene was in between 18 and 41 using the Cepheid`s system. COV2-kit revealed the Ct scores of the same gene as 27–35.5. CDC recommends the upper limit of the Ct as 37. Accordingly, all tested samples were found below this limit by using the COV2-kit approach. In addition, the performance of COV2-kit`s *RdRP* gene *versus* Cepheid`s *N2* gene was compared. Accordingly, the Ct value of the *RdRP* gene (COV2-kit) was found to be lower than *N2* gene (Cepheid`s), which points out the sensitivity of COV2-kit than Cepheid`s system. Nevertheless, the Ct score of three COVID19 patients of *N2* gene and one of *RdRP* gene were out of the upper threshold (Fig 3B). Additionally, multiplex (triplex) rRT-PCR standardization test showed that this assay is efficient to detect three genes in the same reaction tube.

Reverse transcription polymerase chain reaction (RT-PCR) is a sensitive assay for the detection of specified gene sequences encoding the proteins of the virus, such as RNA-dependent RNA polymerase (*RdRP*), nucleocapsid (*N*), envelope (*E*), and spike (*S*). Although RT-PCR tests are widely applied, and substitution tests are being developed, the current testing capability must meet new universal demands for fast, credible, and possible molecular detection [23]. In this method, we tried to bridge many challenges and weakness resulted in former assays and take benefits from their results and applications to improve novel assay. Compared to simplex or duplex analyses, the triplex analysis provides more accuracy and avoids possible negative false results that may be caused by a mutation in one of the viral genes [24]. *E* gene is an important gene codes for the envelope (E) protein of SARS-CoV-2. It is an integral membrane protein functional in several biological processes of the virus such as assembly, budding, envelope formation, and pathogenesis [25]. Additionally, RNA-dependent RNA polymerase (*RdRP*) gene encodes an enzyme responsible for viral replication. These indispensable genes are the main target for the molecular detection of SARS-CoV-2 [12, 26–29]. Kakhki et al. [30] reported that *ORF8* gene is a possible target for detecting COVID-19 and it is different from reported genes (*RdRP*, *E* and *N* genes), and ORF8 is fully specific to COVID-19 and has no cross-reactivity with other types of coronaviruses. They reported that *ORF8* gene can be considered as a supplemental confirmatory test.

Our study partly agrees with a protocol of a study published by Park et al. [31], based on a strategy for styling dynamic primer kits, and nominating reaction status leads to the top utilization of these primers. They reported three-steps guidelines for designing and optimization specific primer sets: 1) the selection of primer sets for target genes (*RdRP*, *N*, *E*, and *S*) in SARS-COV-2 genome, 2) the *in-silico* effectiveness of primer and sequences of the amplicon, and 3) the optimization of PCR conditions. The real-time PCR-dependent protocol was developed to rather than traditional PCR-based protocols and used a multiplex PCR-based protocol permitting the simultaneous amplification of multiple gene targets such as *RdRP*, *N*, *E*, and *S*. This allows to run the reaction in a single tube. The described protocol which is like ours is wide-scale, high-integrity and accepted as gold standard. It will be applied to a larger number of viral extracted clinical RNA samples in subsequent tests to verify the specificity and sensitivity of the study. Although this strategy focused on SARS-CoV-2, but it is also applicable to other viruses.

## Conclusions

This study demonstrates a multiplex rRT-PCR method for the diagnosis of SARS-CoV-2. Simultaneous targeting of two viral genes (*RdRP* and *E*) and one human gene (*RP*) in the same PCR reaction provides an accurate, reliable, and easy-to-use SARS-CoV-2 diagnostic test. Additionally, the assay allows working with large numbers of patients using less PCR reagent. Therefore, the cost and reliability of the assay are improved. Although the primer/probe sequences are designed to minimize the possibility of cross-reactivity with other coronavirus strains, they should be tested with emerging mutant variants. The test must be validated using different RT-PCR instruments and can be used in infection prevention and control organizations, hospitals, medical centers, and diagnostic laboratories.
